# Engineering Properties of Engineered Cementitious Composite and Multi-Response Optimization Using PCA-Based Taguchi Method

**DOI:** 10.3390/ma12152402

**Published:** 2019-07-28

**Authors:** Junfei Zhong, Jun Shi, Jiyang Shen, Guangchun Zhou, Zonglin Wang

**Affiliations:** 1 School of Transportation Science and Engineering, Harbin Institute of Technology, Harbin 150090, China; 2 Key Lab of Structures Dynamic Behavior and Control of the Ministry of Education, Harbin Institute of Technology, Harbin 150090, China; 3 Key Lab of Smart Prevention and Mitigation of Civil Engineering Disasters of the Ministry of Industry and Information Technology, Harbin Institute of Technology, Harbin 150090, China

**Keywords:** engineered cementitious composite (ECC), multi-response optimization, Taguchi method, principal component analysis, principal performance

## Abstract

The engineered cementitious composite (ECC) mixtures were prepared with Portland cement, ground fly ash, silica sand, and polyvinyl alcohol (PVA) fibers. Accordingly, four mix design factors with five levels each were designed using the Taguchi method. The engineering properties of ECC (flow expansion, compressive strength, flexural strength, charge passed, and maximum freeze–thaw cycle) were evaluated, and the single-response optimizations were conducted separately. Unlike other studies assigning a relative weighting parameter to each response, the principal component analysis (PCA) was innovatively introduced to optimize the ECC’s multiple responses so that the single principal performance was obtained from the most objective perspective. Furthermore, the weighting parameters for utility concept were determined by the PCA. Thereafter, an optimum mix formulation was estimated using the PCA-based Taguchi method and the updated utility concept, which provided the most desired balance of these engineering properties. Finally, the contribution of each mix design factor to the principal performance of ECC was examined, and the estimated mix formulation was verified via an additional experiment.

## 1. Introduction

It is well known that discrete fibers can compensate for the shortcomings of ordinary cement-based materials, such as the brittleness, low tensile strength, limited deformability, and cracking resistance. Fiber-reinforced concrete (FRC), ultra-high-performance concrete (UHPC), and engineered cementitious composites (ECC) generally benefit more than ordinary cement-based materials from various fiber reinforcements. Recent investigations further indicated that polyvinyl alcohol (PVA) fibers could effectively promote the ductility and toughness of ECC, leading to its wide application [1,2,3]. The excellent dispersibility in fresh cementitious composites and unique bonding properties of PVA fibers are not matched by other fibers such as polypropylene fibers, polyethylene fibers, and steel fibers [4]. The cementitious composites reinforced with PVA fibers exhibit high deformation capacity and pseudo strain-hardening characteristics [5]. On the other hand, fly ash attracted increasing attention in recent years for its eco-friendly and cement-economizing properties. Higher ductility and smaller crack width are obtained from ECC materials incorporating PVA fibers and fly ash. The ultimate tensile strain of the ECC can be raised to 3–7%, and all crack widths can be controlled to be less than 0.06 mm [6]. However, the effectiveness of fly ash is normally expressed at a relatively late age due to its slow reactivity [7,8]. Recent efforts to deal with the shortcomings of fly ash suggested that the finer particles of fly ash contribute more to the mechanical and durability properties, as the reduced particle size means that the pozzolanic reaction is accelerated [9,10,11,12].

On account of these facts, this investigation utilized PVA fiber and ground fly ash in order to produce high-performance cementitious composites. The mix design procedure involving multiple independent variables was implemented based on the Taguchi method [13]. As a systematic method to reveal the optimum or near-optimum combinations of factors by a small number of experimental runs, the Taguchi method provides the balance of efficiency and cost [14,15]. However, the application of the Taguchi method alone is confined to single-objective processes. It does not match with the majority of cases where multi-response problems arise. In severe environments such as the northeastern region of China, vehicle loads, chloride corrosion, and frost damage are common negative actions which affect the application of ECC materials. The major challenge is to prepare ECC materials of outstanding fresh mix rheology and mechanical strength, as well as resistance to chloride penetration and freeze–thaw cycles. In this investigation, several responses of ECC materials subjected to different performance evaluation tests were simultaneously collected. The precondition for determining the desirable mix formulations was the optimization of these responses.

So far, the desirability function [16,17], genetic algorithm [18,19], response surface methodology [20,21], and utility concept [22,23] were presented for multi-response optimization. These optimization techniques rely on either the assumption that the responses are independent, or engineering experience to assign weighting factors. However, the different performance characteristics of ECC materials have varying degrees of correlations, and the assignment of weights varies in different cases. To overcome these drawbacks, this investigation makes full use of principal component analysis (PCA) to transform possibly correlated responses into uncorrelated principal components. PCA is an effective technique for identifying patterns in correlated data and for expressing the data to highlight their similarities and differences [24]. The principle of PCA is detecting the original data and then compressing them into principal components of fewer dimensions. Thus, the sophisticated multivariate problems can be simplified from the most informative viewpoint. It is a matter of determination as to how many components should be chosen through the statistical analysis. One of the most common criteria proposed in the existing literature for screening the principal components is based on the desired precision. The desired precision is usually between 80% and 90% of the total variance [25]. The cumulative percentages of variance owned by the selected principal components should not be less than the desired precision. Another criterion is Kaiser’s rule which preserves the principal components with an eigenvalue greater than one [26]. Those principal components with eigenvalues less than one are recommended to be filtered out because they contain redundant information [27]. Typically, Kaiser’s rule is considered for formal criteria; thus, it is frequently used to select the principal components. The precision of these principal components can be subsequently assessed by calculating their cumulative percentages of variances. In this regard, the difficulties in unifying the diverse measurement units, identifying the relationships among variables, and assuming the weights can be addressed with the support of PCA. Thereafter, the optimum set of mix design factors can be achieved by applying the Taguchi method to the single response. Although the combination of PCA and the Taguchi method attracted much interest in industrial manufacturing process [28,29,30], it was seldom applied to the preparation of cementitious composites for improving the multiple engineering properties. The successful practice of the PCA-based Taguchi method in many other multi-response optimization cases demonstrates its potential to find the optimum ECC mix formulation.

## 2. Experimental Work 

### 2.1. Materials

Portland cement (P.O. 42.5) and fly ash were used as the binders. A milling procedure was performed to produce finer particles of fly ash as it is more active in the pozzolanic reaction. The chemical compositions of Portland cement and ground fly ash are summarized in Table 1. The silica sand with particle diameter ranging from 0.075 mm to 0.150 mm was used as the fine aggregate. Additionally, polycarboxylic superplasticizer was also added during the mixing procedure to provide the ECC mixtures with desired fresh mix workability. Discrete PVA fibers of 39 μm diameter and 13 mm length were employed in this investigation. These PVA fibers manufactured by Kuraray Co., Ltd. (Tokyo, Japan) complement high levels of tensile strength (1600 MPa) and elastic modulus (42 GPa) with desired ductility (7% strain at failure in tension).

### 2.2. Mix Design

The mix proportions were designed based on the Taguchi method. The content of fly ash (*FA*), sand-to-binder ratio (*S/B*), water-to-binder ratio (*W/B*), and volume fraction of PVA fiber (*V_PVA_*) were selected as the mix design factors. The combinations of these mix factors with five levels were reduced from 625 (25 × 25) possible trials to 25 mix proportions using an orthogonal array (*L*_25_), as summarized in Table 2. The mass fraction of superplasticizer for each mix proportion is also presented in Table 2.

### 2.3. Test Method

**Figure 1:**
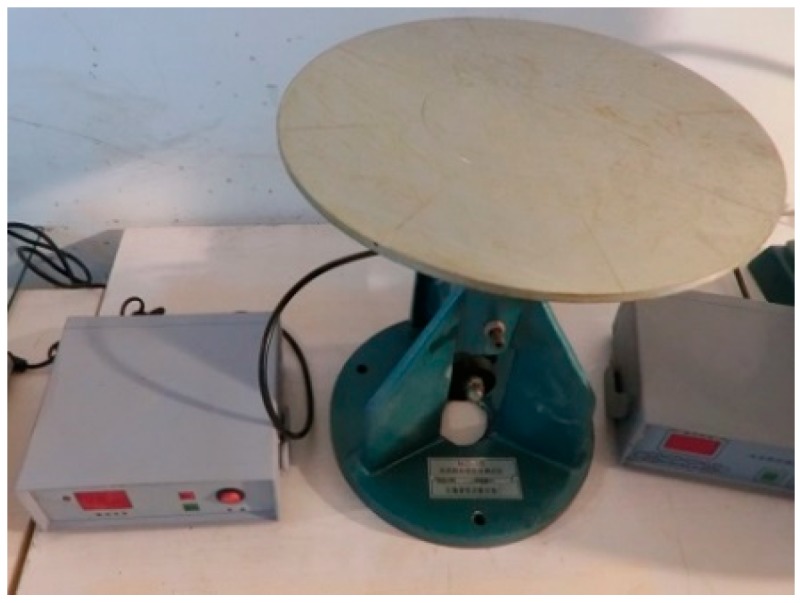
Flow table used in the flow expansion test.

The flow expansion of each mixture was determined per ASTM C230/C230 M [31] to assess the fresh mix rheology of ECC materials. The flow table used in the test is shown in Figure 1. After a flow specimen was spread by the operation of this table, the lengths along two diameters at right angles to each other were measured. The average value was treated as the flow expansion.

**Figure 2:**
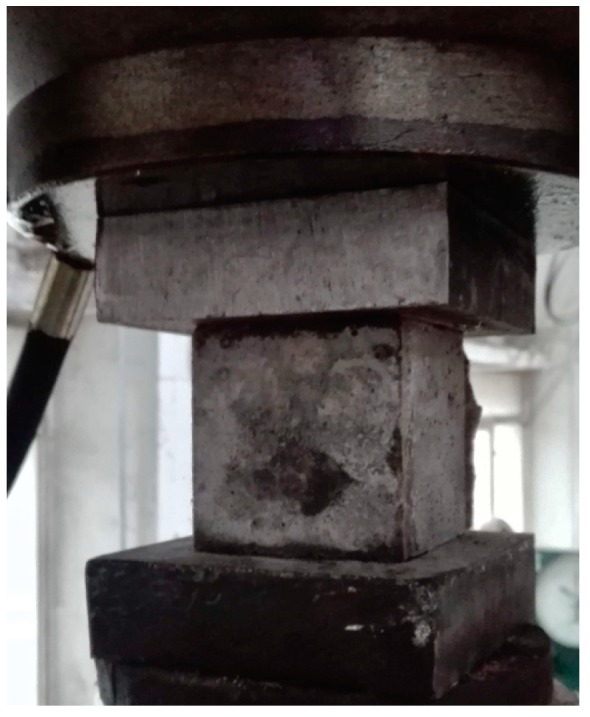
The cubic specimen subjected the compression test.

**Figure 3:**
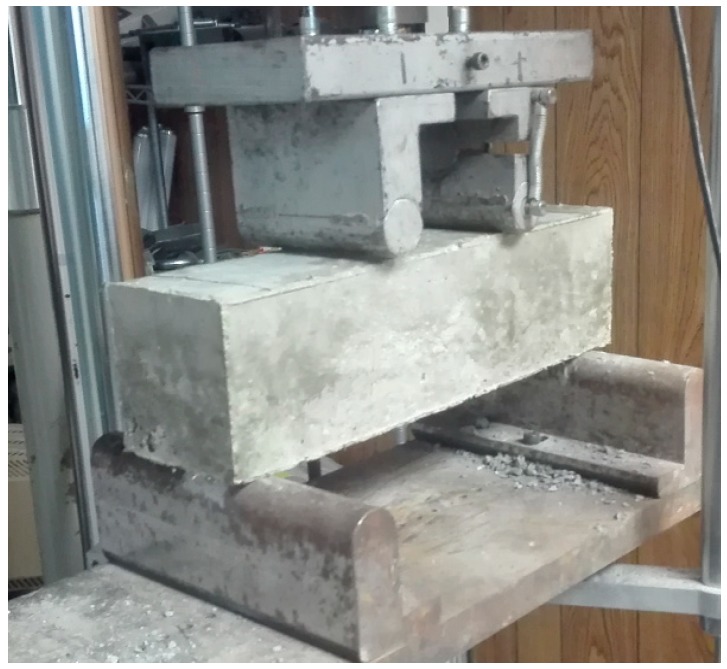
The prismatic specimen subjected to the flexure test.

Cubic specimens 100 mm × 100 mm × 100 mm in size were demolded after 28 days, and then subjected to the compression tests per GB/T 50,081 [32], as shown in Figure 2. The peak load was divided by the bearing area to calculate the compressive strength. As shown in Figure 3, flexure tests were performed on prismatic specimens 100 mm × 100 mm × 400 mm in size via four-point loading on a span of 300 mm per GB/T 50,081 [32]. A servo valve-controlled hydraulic test system was operated at a displacement rate of 0.02 mm/s. Load and deflection data were collected using a load cell and a displacement transducer connected to a data acquisition system. The flexural stress–deflection curves and maximum flexural stress were obtained.

**Figure 4:**
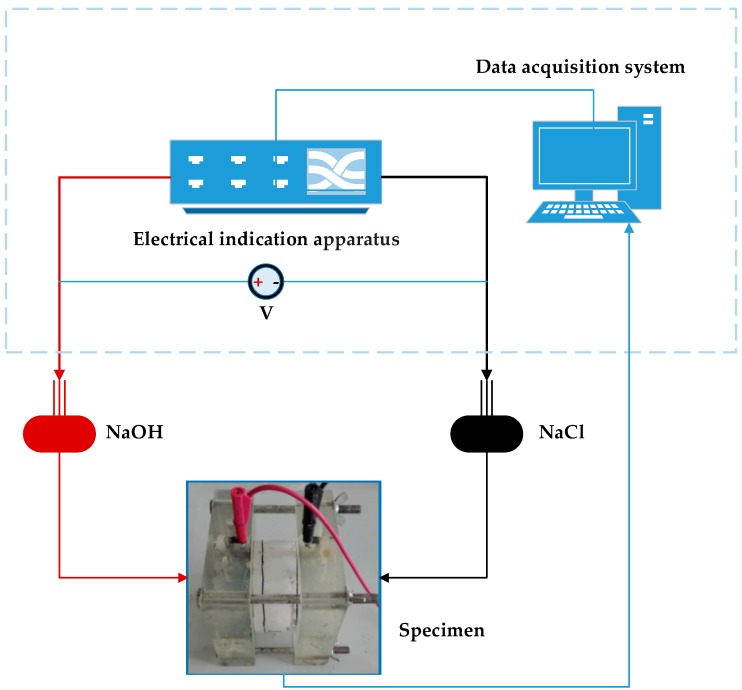
Schematic diagram of the electrical indication test set-up.

The standard test method for electrical indication covers the determination of the electrical conductance of ECC specimens to provide a rapid indication of their resistance to the penetration of chloride ions [33]. The total charge passed during a 6-h period can reflect the ECC’s ability to resist chloride ion penetration. Cylindrical specimens 100 mm in diameter and 50 mm in height were demolded 24 h after casting and then cured for 28 days. The specimens were subjected to the vacuum saturation before electrical indication test. The electrical indication apparatus (NEL-PEU) was used in the test, which maintained a potential difference of 60 V across the ends of a cylindrical specimen. The two ends of the specimen were immersed in a sodium chloride solution (3.0% by mass in distilled water) and a sodium hydroxide solution (0.3 mol/L in distilled water). The electrical current was recorded at 10-min intervals by a data acquisition system. The electrical indication test set-up is illustrated in Figure 4.

**Figure 5:**
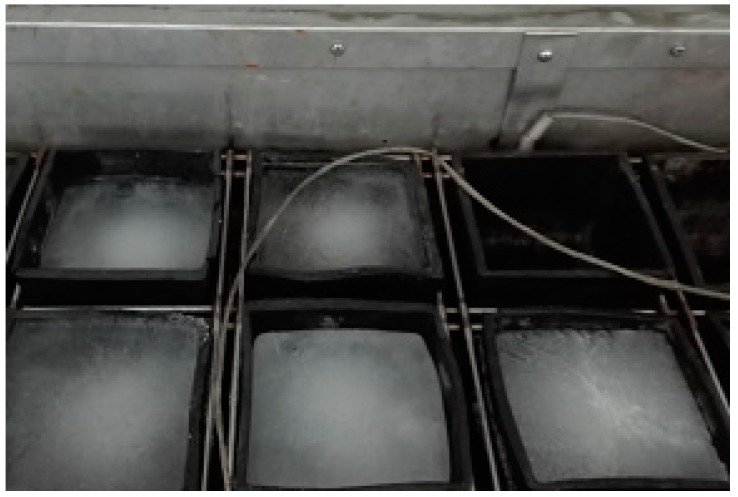
Freeze–thaw test specimens placed in the chamber.

A rapid determination of the ECC’s frost resistance was performed on prismatic specimens (100 mm × 100 mm × 400 mm) per GB/T 50082-2009 and ASTM C666 [34,35]. Specimens were cured for 24 days and then subjected to the vacuum saturation for four days. The test was performed using the rapid freezing-and-thawing apparatus (TDR-1) consisting of a chamber in which the specimens were subjected to automatic and reproducible freeze–thaw cycles produced by the refrigerating and heating procedures, as shown in Figure 5. The fundamental transverse frequency tests were carried out, and the relative dynamic modulus of elasticity (RDME) was calculated after every 25 cycles to detect the internal damage of ECC specimens. The freeze–thaw test was terminated when the RDME dropped below 60%, and the maximum freeze–thaw cycles were recorded.

## 3. Results and Discussion

### 3.1. Engineering Properties of ECC

The Taguchi method uses the signal-to-noise (S/N) ratio as the statistical measure of performance. The S/N ratio depends on the original performance characteristics and generally presents as three types, i.e., nominal-the-best, smaller-the-better, and larger-the-better [15]. Since the higher observed values indicate the better fresh mix rheology, compressive strength, flexural strength, and freeze–thaw resistance, larger-the-better is suitable for measuring these engineering properties. On the contrary, the charge passed is expected to be as low as possible. Hence, smaller-the-better is used to measure the chloride ion penetration resistance of the ECC. The two types of S/N ratios are defined as follows:

(1) $$\text{Larger-the-better},\;\;\;\;\;S/N=-10\times \log_{10}(\frac{1}{n}\sum_{i=1}^{n}\frac{1}{y_{i}^{2}}),$$

(2) $$\text{Smaller-the-better},\;\;\;\;\;S/N=-10\times \log_{10}(\frac{1}{n}\sum_{i=1}^{n}y_{i}^{2}),$$

where *n* indicates the number of observations and *y* represents the results of the observed data. 

**Figure 6:**
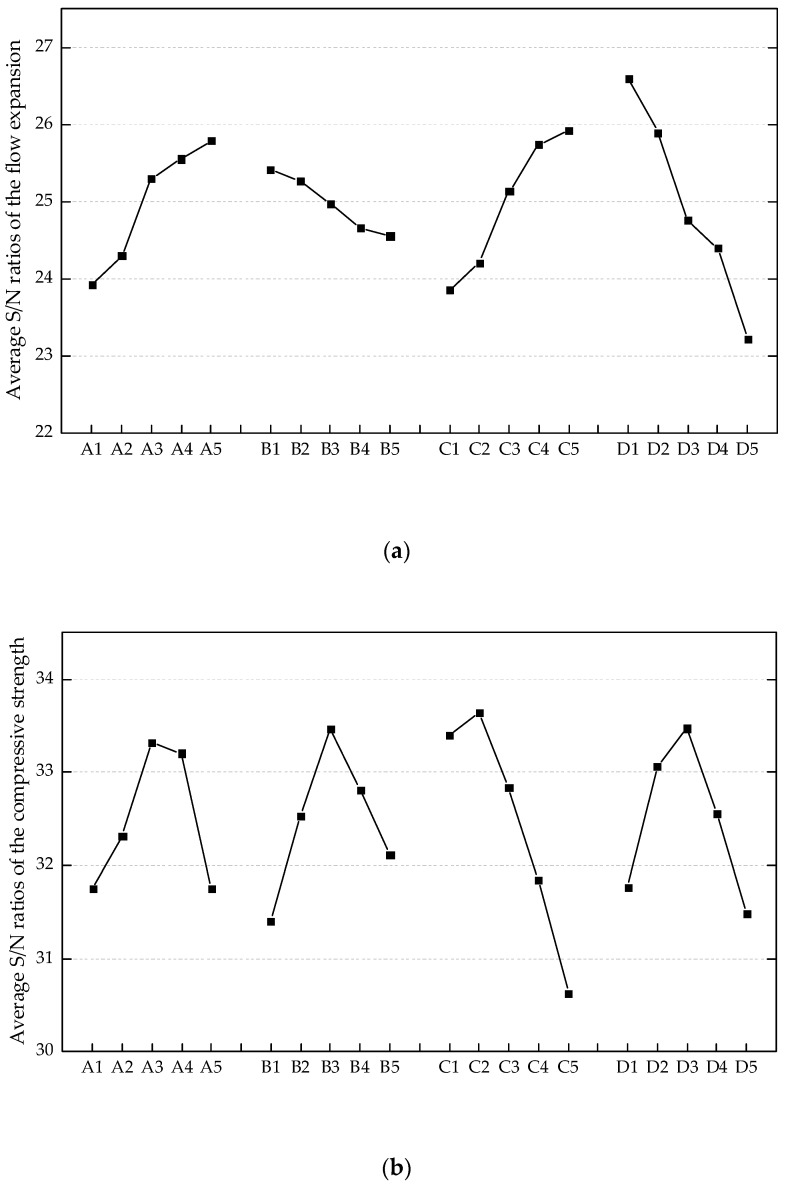

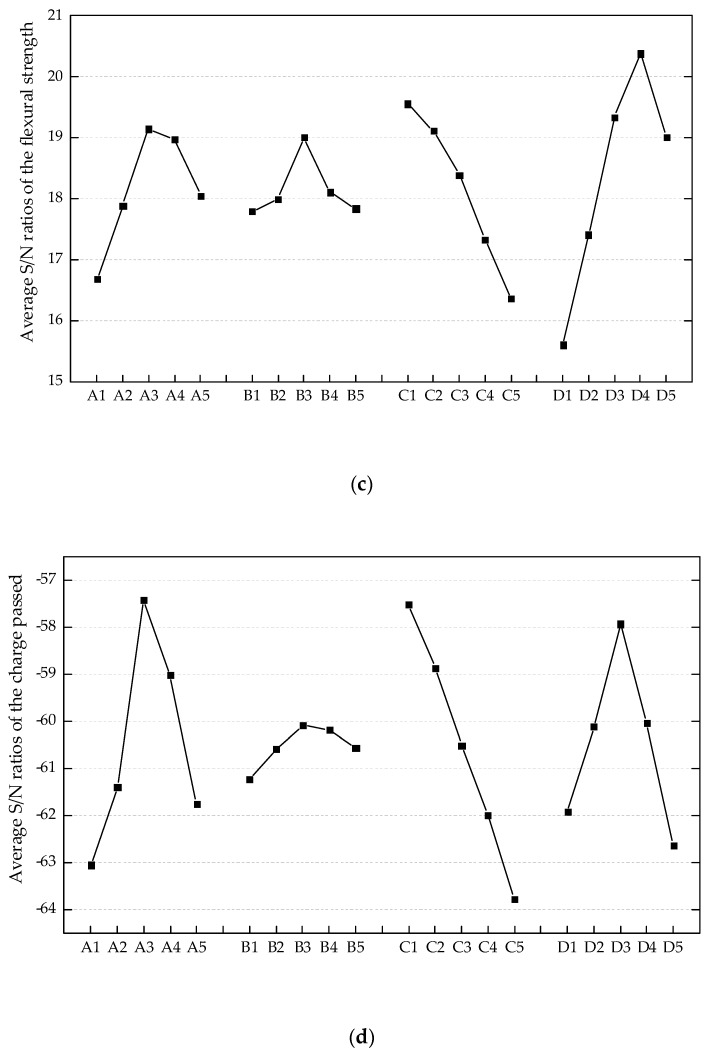

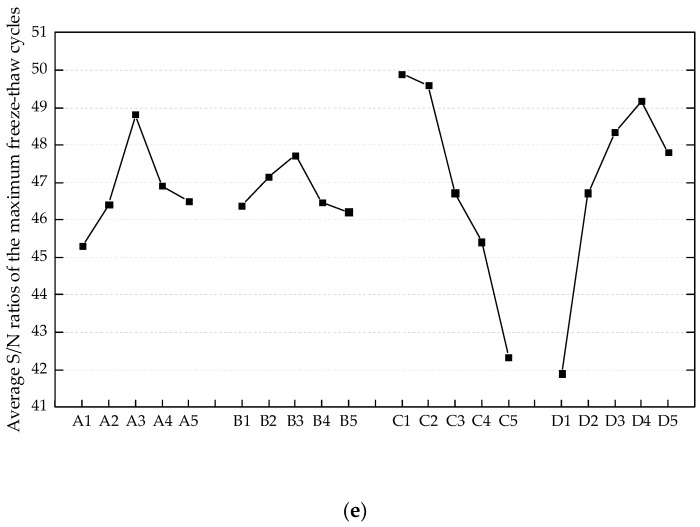
Average signal-to-noise (S/N) ratios of different engineering properties: (**a**) flow expansion; (**b**) compressive strength; (**c**) flexural strength; (**d**) charge passed; (**e**) maximum freeze–thaw cycles.

After the original performance characteristics were transformed into the S/N ratios, their average values at different levels of each factor were calculated. For example, the average S/N ratio at A1 was the arithmetic mean of those ratios for Mix. A1B1C1D1, Mix. A1B4C3D2, Mix. A1B2C5D3, Mix. A1B5C2D4, and Mix. A1B3C4D5. The optimal levels of individual mix design factors were obtained with the highest average S/N ratios. The dependence of average S/N ratios on the varying factors and levels is plotted in Figure 6. 

It is evident from Figure 6a that the flow expansion increases with the ascending *FA* and *W/B*, while simultaneously decreasing with the increasing *S/B* and *V_PVA_*. Despite the possible shape change in fly ash particles resulting from the milling procedure [36], the degeneration of flowability is not observed for ECC mixtures containing ground fly ash. The possible reason is the higher packing density brought about by the ground fly ash that releases the water to lubricate the particles [37,38]. Meanwhile, the micro fly ash particles tend to bind with water, leading to the formation of compounds with a larger volume than the water itself. Therefore, the inter-particle spacing between the rough cement grains is increased [39]. The maximum flow expansion was obtained with A5B1C5D1 (*FA*: 0.700, *S/B*: 0.250, *W/B*: 0.5000, *V_PVA_*: 0).

**Figure 7:**
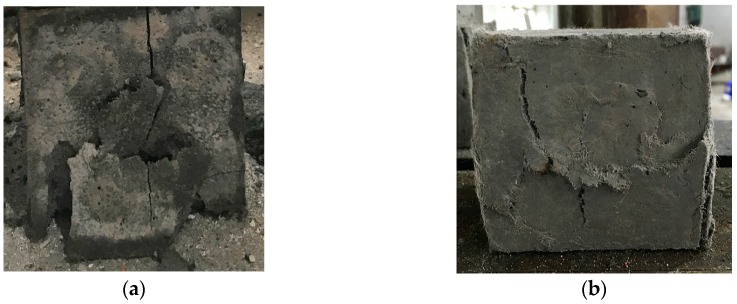
Examples of failed engineered cementitious composite (ECC) compression test specimens with different polyvinyl alcohol (PVA) fiber reinforcement conditions: (**a**) without fiber; (**b**) 1.0 vol.% PVA fiber.

The positive effect of ground fly ash and PVA fibers with proper content (*FA*: 0.175–0.525; *V_PVA_*: 0.005–0.015) on the compressive strength is exhibited in Figure 6b. The most significant improvement in the compressive strength was achieved with *FA* ranging from 0.375 to 0.525, which could be the result of relatively early pozzolanic reaction between the fly ash and calcium hydroxide. In terms of the PVA fiber reinforcement, the bridging effect acts as the lateral constraint for the specimens subjected to the vertical loading. While the ECC specimen split into parts and experienced spalling due to the absence of fibers (Figure 7a), the specimen reinforced with PVA fibers did not disintegrate even at the stage of failure in compression (Figure 7b). However, there is a risk of strength degradation resulting from the increase in fiber content, as shown in Figure 6b. The pores induced by excessive fibers are likely to worsen the density of specimens. Additionally, the compressive strength of ECC can obviously benefit from the moderate *S/B* (around 0.500) and low *W/B* (0.2500–0.3125). The maximum compressive strength was obtained with A3B3C2D3 (*FA*: 0.350, *S/B*: 0.500, *W/B*: 0.3125, *V_PVA_*: 0.010).

**Figure 8:**
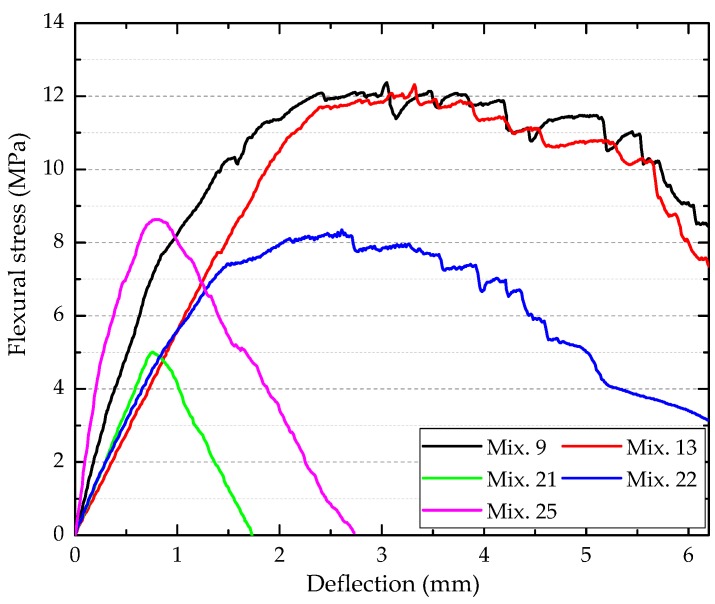
The flexural stress–deflection curves of typical ECC mixtures.

As shown in Figure 6c, the low *W/B* (0.2500–0.3125), moderate *FA* (0.350–0.525), and *S/B* (around 0.500) favored the flexural strength of ECC. In addition, the flexural strength was improved by 0.5–2 vol.% PVA fiber reinforcement. It was attributed to the notable role of PVA fiber in mitigating micro-cracking. The fibrous ECC specimens (e.g., Mix. 9, 13, 22, and 25) exhibited superior load capacity and energy dissipation capacity (areas underneath the flexural stress–deflection curves) to the plain specimens (e.g., Mix. 21), as shown in Figure 8. Nevertheless, excessive fibers are detrimental to the flowability and density as discussed above. Consequently, the flexural strength decreased when *V_PVA_* > 1.5% (Figure 6c) and the relatively poor post-cracking performance of Mix. 25 was observed (Figure 8). The maximum flexural strength was obtained with A3B3C1D4 (*FA*: 0.350, *S/B*: 0.500, *W/B*: 0.2500, *V_PVA_*: 0.015). 

By the characteristics of average S/N ratios shown in Figure 6d, the resistance to chloride ion penetration was greatly enhanced around *FA* = 0.375. The fine fly ash can make up for the defect of pore structures. Another promotion for the impermeability was found in the presence of PVA fibers with 0.5–1.5 vol.%. The specific surface properties of PVA fibers facilitate their dispersion in fresh cementitious composites and bonding with the matrix. Therefore, a stable reinforcement is formed inside the ECC materials which can bridge the micro-cracks and control the crack width. It should be noted that the chloride ion penetration is very sensitive to the volume fraction of PVA fiber. The decline in S/N ratio was observed at *V_PVA_* = 2.0% (lower than the S/N ratio at *V_PVA_* = 0) due to the difficulties in providing a homogeneous distribution of numerous fibers in the ECC mixtures. Moreover, the ECC became obviously more permeable with the ascending *W/B*. Both the hydration progress and the evaporation of free water induce some deficiencies in the paste and the interfaces, thereby weakening the resistance to chloride ion penetration. It can be observed from Figure 6d that the A3B3C1D3 (*FA*: 0.350, *S/B*: 0.500, *W/B*: 0.2500, *V_PVA_*: 0.010) provided the minimum amount of charge passed. 

As shown in Figure 6e, it is explicit that the freeze–thaw resistance faded with the increasing *W/B*. Extensive mixing of water normally leads to a porous internal structure. The maximum freeze–thaw cycles increased drastically as a result of PVA fiber reinforcement. The air-entraining and pressure-release effects of PVA fibers inhibit the development of micro-cracks and enable ECC specimens to work under the frost exposure for a relatively long period [40]. The volume fraction of PVA fibers should be well controlled as the reduction in S/N ratio was observed at *V_PVA_* = 2.0%. Reasonable *S/B* also helps the ECC materials gain adequate density and strength prior to the frost exposure. Nevertheless, higher sand content is against the freeze–thaw resistance since the paste ratio is simultaneously decreased. Furthermore, the ground fly ash was found to be beneficial to the freeze–thaw resistance, especially at a replacement rate of 35%. The optimum condition under the frost exposure was A3B3C1D4 (*FA*: 0.350, *S/B*: 0.500, *W/B*: 0.2500, *V_PVA_*: 0.015).

### 3.2. Principal Component Analysis of Test Data

Five engineering properties were evaluated before the multi-response optimization. The optimum mix formulations for these responses were not coincident. Principal component analysis (PCA) was introduced as a powerful statistical method to convert the original responses into a set of lower-dimensional variables. These variables were the preserved principal components carrying the most amount of original information. During the decision-making process, those principal components accounting for most of the variance were prioritized. In the application of PCA, all the S/N ratios were arranged into the initial multi-response array {*M*}:

(3) $$M=\{R_{1},R_{2},\ldots ,R_{p}\}=\left\{\begin{array}{ccc} R_{1,1} & \cdots & R_{1,p}\\ \vdots & \ddots & \vdots \\ R_{q,1} & \cdots & R_{q,p} \end{array}\right\},$$

where *R_i,j_* (*i* = 1, 2, *…*, *q*; *j* = 1,2, *…*, *p*) is the S/N ratio of the *j*th response collected from the *i*th trial, *q* is the number of trials under each test condition, and *p* is the number of responses. The S/N ratios were normalized as follows to eliminate the difference among units:

(4) $$N_{i,j}=\frac{R_{i,j}-R_{j,min}}{R_{j,max}-R_{j,min}},$$

where *N_i,j_* is the normalized S/N ratio, *R_j_*_,max_ is the maximum S/N ratio of the *j*th response, and *R_j_*_,min_ is the minimum S/N ratio of the *j*th response. Thus, the normalized multi-response array can be expressed by

(5) $$N=\{N_{1},N_{2},\ldots ,N_{p}\}=\left\{\begin{array}{ccc} N_{1,1} & \cdots & N_{1,p}\\ \vdots & \ddots & \vdots \\ N_{q,1} & \cdots & N_{q,p} \end{array}\right\}.$$

The correlation array {*C*} was generated by calculating the elements *C_l,k_* as follows:

(6) $$C_{l,k}=\frac{\mathrm{Cov}(N_{i,l},N_{i,k})}{\sqrt{\mathrm{Var}(N_{i,l})\mathrm{Var}(N_{i,k})}},\;l,k=1,\;2,\;\ldots \;,\;p,$$

(7) $$C=\left\{\begin{array}{ccc} C_{1,1} & \ldots & C_{1,p}\\ \vdots & \ddots & \vdots \\ C_{p,1} & \ldots & C_{p,p} \end{array}\right\},$$

where Cov(*N_i,l_, N_i,k_*) denotes the covariance of *N_i,l_* and *N_i,k_*; Var (*N_i,l_*) and Var (*N_i,k_*) are the variance of *N_i,l_* and *N_i,k_*, respectively. The eigenvalues and eigenvectors can be defined as follows:

(8) (C−λE)V=0,

where *λ* and [*V*] = [*a_1_*, *a_2_*, …, *a_p_*]^T^ refer to the eigenvalues and eigenvectors of correlation array {*C*}, respectively. The *j*th eigenvector [*V_j_*] = [*a_1j_*, *a_2j_*, …, *a_pj_*]^T^ meets the condition (*a_1j_*)^2^ + (*a_2j_*)^2^ + … + (*a_pj_*)^2^ = 1. The *j*th principal components [*P_j_*] are formulated as

(9) $$P_{j}=\sum_{l=1}^{p}a_{lj}\times N_{l}.$$

**Figure 9:**
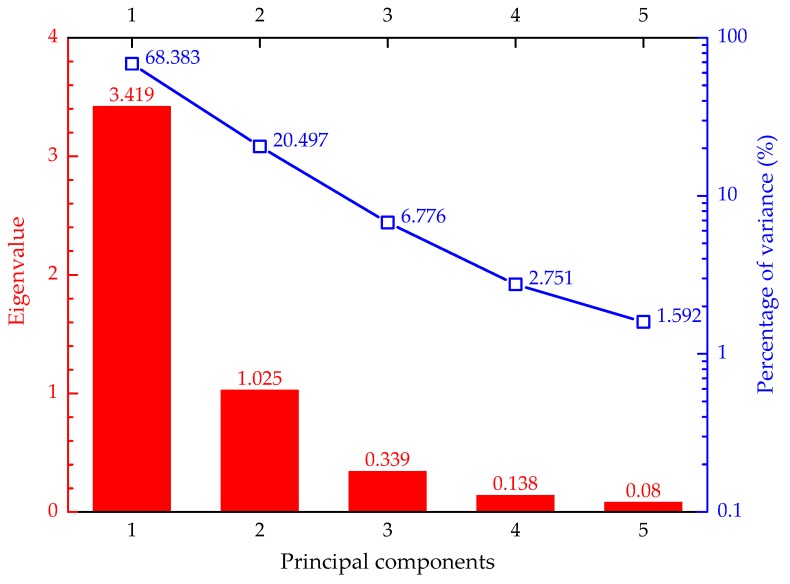
The principal components with their eigenvalues and percentages of variances.

Given *q* observations with *p* responses to be analyzed, then the number of acquired principal components is min (*q − 1*, *p*). All the eigenvalues add up to the number of responses. It should be noted that the sum of the variances remains unchanged through the transformation from the original responses into principal components. The percentage of variance owned by each principal component interprets its relative role. These principal components were ranked in such a way that the first principal component had the largest eigenvalue/percentage of variance, and each succeeding component, in turn, had a descending eigenvalue/percentage of variance, as presented in Figure 9. 

**Figure 10:**
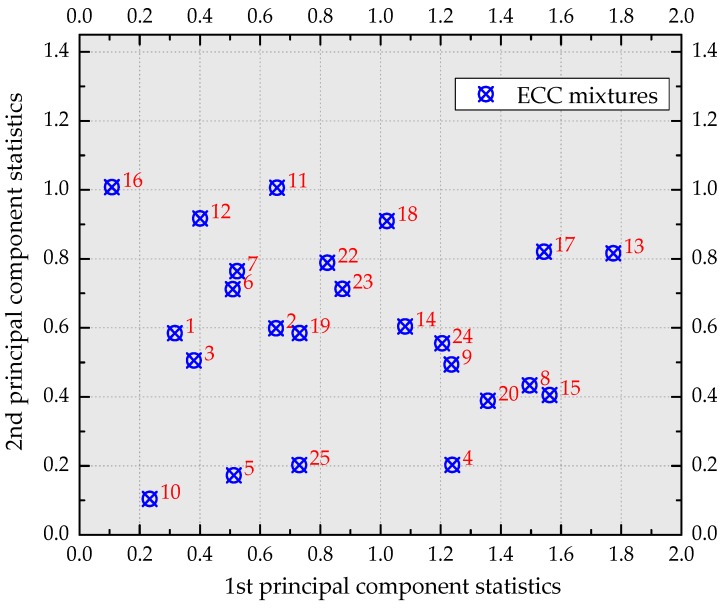
The distribution of ECC mixtures in the two-dimensional space represented by the top two principal components.

Differing from the possibly correlated initial variables, each component was not affected by the others. The five principal components were the pending targets for the multi-response optimization. Hence, it was a matter to determine which principal component should be selected. Based on Kaiser’s rule, the principal components with eigenvalues greater than one were meaningful. Consequently, the initial five-dimensional responses were reduced into the current principal components of two dimensions (first and second principal components). The top two principal components in lieu of the original responses kept the most useful information since they had the predominant percentage of variance (88.88% in total). The eigenvectors for the first and second principal components were [−0.298, 0.449, 0.489, 0.456, 0.513] and [0.764, 0.400, −0.179, 0.454, −0.139], respectively. The elements of each eigenvector were the coefficients for the mathematical function transforming the original responses into the corresponding principal components. Figure 10 exhibits the distribution of numbered ECC mixtures in the space represented by the top two principal components. Actually, the principal component could be regarded as the “generalized engineering property” of ECC materials. Therefore, the difference in the performance characteristics could be embodied by the two principal components.

These two principal components needed to be integrated into a comprehensive performance characteristic index for further analysis. The term coefficient of determination *η* was defined as follows [28]:

(10) $$\eta_{j}=\frac{\lambda_{j}}{p},\;j=1,2,\ldots ,p.$$

**Figure 11:**
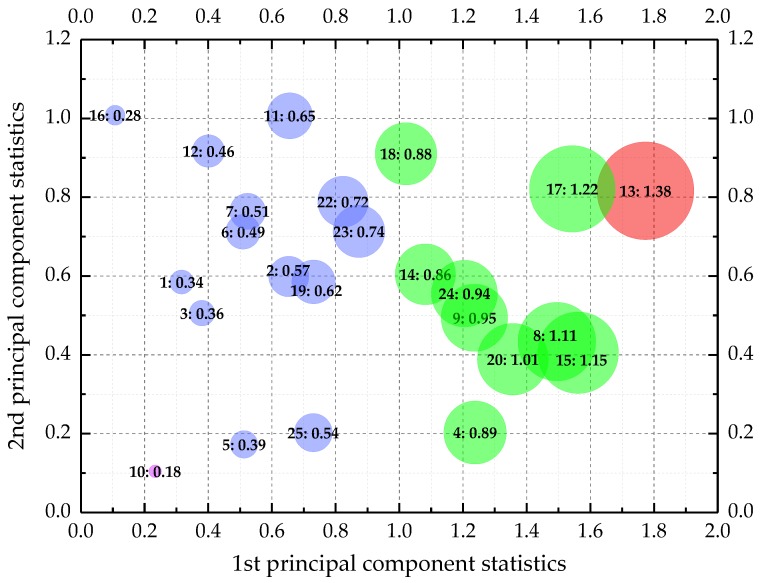
The principal performance statistics of ECC mixtures.

Then, the comprehensive performance characteristic index was equal to the sum of the principle component statistics multiplied by the coefficient of determination.

(11) $$P=\sum_{j=1}^{n}\eta_{j}P_{j},\;n\leq p,$$

where *n* is the number of the selected principal components. Considering that the index features the performance of ECC materials under the influence of multiple test conditions, it was defined as the principal performance herein. The principal performance embodied the essential integration of the original engineering properties. Figure 11 is a bubble chart which vividly exhibits the unitless principal performance statistics of the 25 ECC mixtures. All the bubbles distributed in the two-dimensional space were labeled as “the number of mixtures: the corresponding principle performance statistics”.

### 3.3. Estimation of the Optimum Mix Formulation

**Figure 12:**
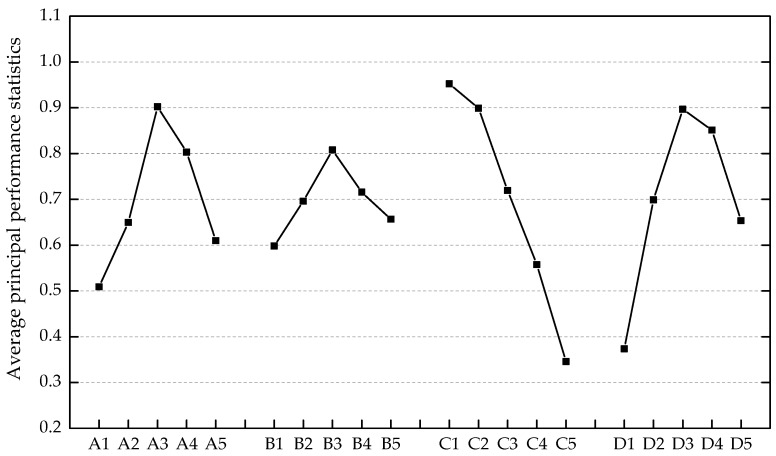
The dependence of average principal performance statistics on varying factors and levels.

The multiple engineering properties were merged into the single principal performance covering the fresh property, the hardened property, and the durability property of ECC materials. The optimum mix formulation reaching the highest average principal performance statistics of each mix design factor was deemed to provide the most desired balance of the five basic engineering properties. The average principal performance statistics at different levels of each mix design factor were calculated, and the results are presented in Figure 12. Except for the monotonic influence of *W/B* on the principal performance, turning points existed in the relationships between principal performance statistics and the other factors (*FA*, *S/B*, and *V_PVA_*). It was found that the combination of A3 (*FA* = 0.350), B3 (*S/B* = 0.500), C1 (*W/B* = 0.2500), and D3 (*V_PVA_* = 0.010) constituted the optimum mix formulation.

As an approach frequently used in the multi-response optimization for cement-based materials [41,42,43], the utility concept was also applied to the multivariate analysis for comparison. In this case, the preference numbers were calculated to measure the original responses, and the overall response (utility value) was the sum of weighted preference numbers [44]. However, the ideal weighting values for responses are usually based on assumption, thereby leading to a certain amount of randomness. To address this issue, the coefficients and eigenvalues deduced by PCA were used. Considering that (*a_1j_*)^2^ + (*a_1j_*)^2^ + … + (*a_pj_*)^2^ = 1, the weighting parameter for the *l*th response (*ω_l_*) could be expressed by a simple linear equation containing the squares of elements from the top two principal components’ eigenvectors. Thus, the *ω_l_* was formulated as follows:

(12) $$\omega_{l}=\alpha_{1}a_{l1}^{2}+\alpha_{2}a_{l2}^{2},\;l=1,2,\ldots ,p,$$

where *α*_1_ and *α*_2_ are the coefficients of correction affected by the relative roles of principal components. It should be a good attempt to determine each coefficient using the eigenvalue of the relevant principal component. Since the weights comply with the condition *ω*_1_ + *ω*_2_ + … + *ω_p_* = 1, it can be concluded that *α*_1_ + *α*_2_ = 1. Thus, the coefficients of correction were calculated as follows:

(13) $$\{\begin{array}{c} \alpha_{1}=\frac{\lambda_{1}}{\lambda_{1}+\lambda_{2}}\\ \alpha_{2}=\frac{\lambda_{2}}{\lambda_{1}+\lambda_{2}} \end{array},$$

where *λ*_1_ and *λ*_2_ are the eigenvalues of the first and second principal components, respectively. Thus, the weighting parameter for the *l*th response (*ω_l_*) was established as follows:

(14) $$\omega_{l}=\frac{\lambda_{1}}{\lambda_{1}+\lambda_{2}}a_{l1}^{2}+\frac{\lambda_{2}}{\lambda_{1}+\lambda_{2}}a_{l2}^{2},\;l=1,2,\ldots ,p,$$

where *a_l_*_1_ and *a_l_*_2_ are the elements from the eigenvectors of the corresponding principal components. The results are tabulated in Table 3.

**Figure 13:**
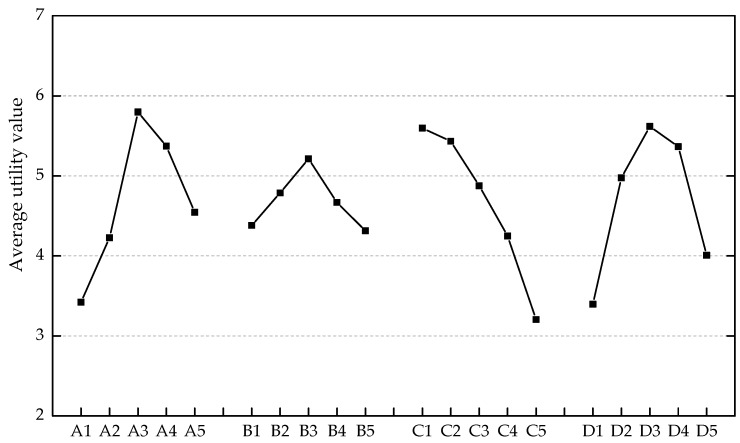
The dependence of average utility values on varying factors and levels.

The average utility values at different levels of each mix design factor were calculated, and the results are presented in Figure 13. The optimum mix formulation was A3B3C1D3 in this case, exactly the same as that obtained from PCA. It could be explained that the inherent information remained through data processing. Additionally, the mathematical expression linking these responses was established via PCA from the most objective perspective. The weighting values were affected by the original test data alone, free of human judgment. Given these reasons, it should be reliable to infer the weighting parameters for the utility concept based on PCA. Furthermore, the PCA shall be of greater accuracy in the multi-response optimization and the weight assignment for utility concept provided that the test data have a larger sample capacity.

### 3.4. Analysis of Variance

The analysis of variance (ANOVA) following the confidence level of 95% was implemented by Minitab 17 to quantify the effect and relative importance of each mix design factor.

**Figure 14:**
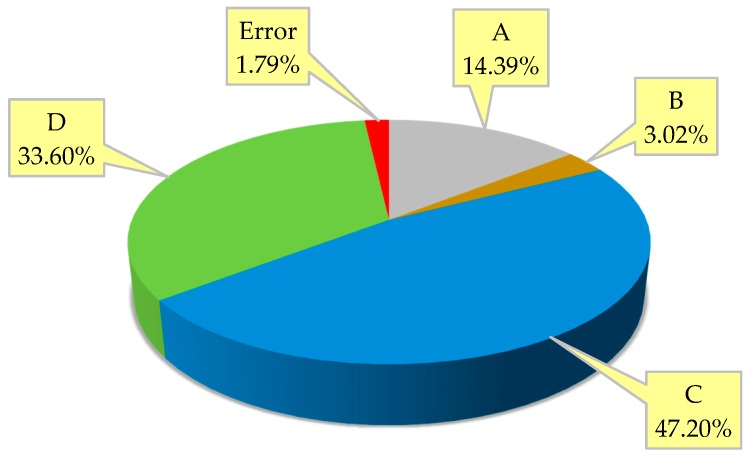
Contribution rates of mix design factors. (A: *FA*; B: *S/B*; C: *W/B*; D: *V_PVA_*.)

As presented in Table 4, the symbol “O” and “X” denote the significant and insignificant factor, respectively. *FA*, *W/B*, and *V_PVA_* were identified as the significant factors, while *S/B* with an *F*-value lower than the critical level (3.84) was insignificant. Furthermore, the contribution rates are plotted in Figure 14, implying the relative importance of each mix design factor. The *W/B* was observed to be the dominant factor governing the principal performance, which accounted for 47.20% of the total contribution. The PVA fiber was indicated to be an effective reinforcement system for the ECC materials, as it contributed up to 33.60%. Moreover, the effect of ground fly ash as a partial substitution for Portland cement cannot be ignored, the contribution rate of which was 14.39%. It can be stated that both PVA fiber and ground fly ash were the indispensable compositions benefiting the ECC’s overall engineering properties.

### 3.5. Confirmation Experiment

It was necessary to verify the estimated optimum mix formulation. Since the optimum mix formulation inferred by the PCA-based Taguchi method was not included in the *L*_25_ orthogonal array, the confirmation experiment was carried out to obtain the actual responses of Mix. A3B3C1D3 (*FA*: 0.350, *S/B*: 0.500, *W/B*: 0.2500, and *V_PVA_*: 0.010). The flow expansion test, compression test, flexure test, electrical indication test, and cyclic freeze–thaw test were performed on the ECC specimens prepared following A3B3C1D3. The test results were compared with the estimated value computed by Equation (15) [45].

(15) $$Q_{E}=\bar{Q}+({\bar{A}}_{3}-\bar{Q})+({\bar{B}}_{3}-\bar{Q})+({\bar{C}}_{1}-\bar{Q})+({\bar{D}}_{3}-\bar{Q}),$$

where $\bar{Q}$ is the mean of the experimental performance characteristic statistics, $\bar{A}$*_3_*, $\bar{B}$*_3_*, $\bar{C}$*_1_*, and $\bar{D}$*_3_* are the average experimental performance characteristic statistics at the optimal levels of mix design factors, and *Q_E_* is the estimated value of the performance characteristic. Assuming the confidence level of 95%, the confidence interval could be calculated by Equation (16) [45].

(16) $$CI_{CE}={\left[F_{0.05}(1,f_{e})V_{e}((1+T_{dof})/N+(1/S))\right]}^{\left(1/2\right)},$$

where *F*_0.05_*(1, f*_e_*)* is the *F*-value corresponding to the confidence level of 95%, and *f*_e_ refers to the errors’ degrees of freedom; *V*_e_ is the variance of errors; *S* is the number of replications for confirmation experiments, *N* is the total number of experiments, and *T*_dof_ is the total degrees of freedom related to the estimated value. The estimated values and verified results are summarized in Table 5. It can be seen that all of the experimental values were within the estimated ranges.

## 4. Conclusions

In this investigation, the Taguchi method combined with PCA was used as the first attempt to extract the most useful information from the multivariate test data, thereby accomplishing multi-response optimization for ECC materials. Material testing and statistical analysis were conducted to reach the following conclusions:

1. The original five engineering properties, including flow expansion, compressive strength, flexural strength, charge passed, and maximum freeze–thaw cycles, can be integrated into the single principal performance by the PCA without loss of important information. The principal performance embodies the essential integration of the original responses.
2. A new approach based on the PCA was devised to help determine the weighting parameters for utility concept. The optimization results obtained from the updated utility concept were consistent with the PCA-based Taguchi method.
3. The analyses of each engineering property and the principal performance indicated that PVA fibers and ground fly ash with proper content (*V_PVA_*: 0.010–0.015; *FA*: 0.350–0.525) can significantly improve the fresh, hardened, and durability properties of ECC materials. Moreover, the analysis of variance points to the considerable contribution of PVA fiber reinforcement (33.60%) to the principal performance.
4. An optimum ECC mix formulation (*FA*: 0.350, *S/B*: 0.500, *W/B*: 0.2500, and *V_PVA_*: 0.010) is recommended through statistical analysis of the principal performance. This mix formulation provides the most desired balance of flowability, compressive strength, flexural strength, chloride ion penetration resistance, and freeze–thaw resistance, which was verified by the additional experiment. This hybrid method provides a reliable reference for the ECC’s multi-performance-oriented mix design.

## Figures and Tables

**Table 1:** Chemical compositions of binders.

Hydraulic Binders	Chemical Analysis of Basic Oxides (wt. %)					
	SiO_2_	Al_2_O_3_	Fe_2_O_3_	CaO	MgO	SO_3_
Portland cement	21.08	5.47	3.96	62.28	1.73	2.63
Ground fly ash	55.70	25.63	5.65	6.93	2.25	0.60

**Table 2:** The mix proportions of engineered cementitious composites (ECCs) using the *L*_25_ orthogonal array. *FA*—fly ash content; *S/B*—sand-to-binder ratio; *W/B*—water-to-binder ratio; *V_PVA_*—volume fraction of polyvinyl alcohol.

Mixture	Labels	Factors & Levels				Superplasticizer (%)
		A(*FA*)	B(*S/B*)	C(*W/B*)	D(*V_PVA_*)	
1	A1B1C1D1	0	0.250	0.2500	0	0.82
2	A1B4C3D2	0	0.625	0.3750	0.005	0.38
3	A1B2C5D3	0	0.375	0.5000	0.010	0.09
4	A1B5C2D4	0	0.750	0.3125	0.015	1.07
5	A1B3C4D5	0	0.500	0.4375	0.020	0.94
6	A2B4C2D1	0.175	0.625	0.3125	0	0.22
7	A2B2C4D2	0.175	0.375	0.4375	0.005	0.05
8	A2B5C1D3	0.175	0.750	0.2500	0.010	1.51
9	A2B3C3D4	0.175	0.500	0.3750	0.015	0.30
10	A2B1C5D5	0.175	0.250	0.5000	0.020	0.50
11	A3B2C3D1	0.350	0.375	0.3750	0	0.63
12	A3B5C5D2	0.350	0.750	0.5000	0.005	0.06
13	A3B3C2D3	0.350	0.500	0.3125	0.010	0.48
14	A3B1C4D4	0.350	0.250	0.4375	0.015	0
15	A3B4C1D5	0.350	0.625	0.2500	0.020	1.52
16	A4B5C4D1	0.525	0.750	0.4375	0	0.48
17	A4B3C1D2	0.525	0.500	0.2500	0.005	0.68
18	A4B1C3D3	0.525	0.250	0.3750	0.010	0.10
19	A4B4C5D4	0.525	0.625	0.5000	0.015	0.05
20	A4B2C2D5	0.525	0.375	0.3125	0.020	0.59
21	A5B3C5D1	0.700	0.500	0.5000	0	0
22	A5B1C2D2	0.700	0.250	0.3125	0.005	0.67
23	A5B4C4D3	0.700	0.625	0.4375	0.010	0.04
24	A5B2C1D4	0.700	0.375	0.2500	0.015	1.42
25	A5B5C3D5	0.700	0.750	0.3750	0.020	0.87

**Table 3:** Weighting parameters assigned to each response.

Response	Weighting Parameter
Flow expansion	0.203
Compressive strength	0.192
Flexural strength	0.191
Charge passed	0.207
Maximum freeze–thaw cycles	0.207

**Table 4:** Results of ANOVA for the principal performance.

Mix Design Factor	*DF*	*SS*	*MS*	*F*-Value	Contribution (%)	Significance
A	4	105.75	26.44	16.05	14.39	O
B	4	22.19	5.55	3.37	3.02	X
C	4	346.93	86.73	52.64	47.20	O
D	4	246.92	61.73	37.47	33.60	O
Error	8	13.18	1.65	-	1.79	-
Total	24	734.97	-	-	100.00	-

Notes: *DF*, degree of freedom; *SS*, sum of squares; *MS*, mean of squares.

**Table 5:** Results verified by the additional experiment.

Optimum Combination (A3B3C1D3)	Estimated Value	Experimental Value
Flow expansion (cm)	15.84 ± 3.64	17.00
Compressive strength (MPa)	63.16 ± 8.51	60.50
Flexural strength (MPa)	13.00 ± 1.83	12.17
Charge passed (C)	219.43 ± 281.43	359.77
Maximum freeze–thaw cycles	414 ± 73.99	425
